# The majority of opioid prescriptions in Israel between 2010 and 2020 involved a small minority of physicians and of patients: policy implications

**DOI:** 10.1186/s13584-025-00743-y

**Published:** 2025-12-30

**Authors:** Ehud Kaliner, Matan J. Cohen, Adam J. Rose, Reuven L. Dressler

**Affiliations:** 1 State of Israel Ministry of Health, Central District, 44 HaPalmach St., 9254267 Jerusalem, Israel; 2 Clalit Health Services, Jerusalem District, Hebrew University of Jerusalem Faculty of Medicine, 4 Eli Cohen St., 4723953 Ramat Hasharon, Israel; 3 School of Public Health, Faculty of Medicine, The Hebrew University of Jerusalem, Hebrew University, Ein Kerem Campus, 91120 Jerusalem, Israel; 4 Clalit Health Services, Department of Family Medicine, Hebrew University of Jerusalem Faculty of Medicine, HaGitit 64-B, 9839037 Maale Adumim, Israel

**Keywords:** Opioids, Multiple prescriptions, Overuse

## Abstract

**Background:**

We sought to examine patterns of opioid prescriptions in Israel, focusing on the extent to which patients received prescriptions from multiple physicians and physicians prescribed opioids to few or many patients.

**Methods:**

We conducted a historical cohort analysis using a data repository of Clalit Health Services (CHS), Israel’s largest health maintenance organization (serving about five million individuals), between 2010 and 2020. We included all non-oncological adult patients registered with CHS who received opioids during the study period. We examined the number of physicians prescribing opioids to each patient and the number of patients prescribed opioids per physician. We also assessed the difference in opioid prescription patterns based on whether patients were registered with the prescribing physicians.

**Results:**

During the study period, 868,499 adult patients filled 5,600,598 opioid prescriptions. Half of the patients received opioid prescriptions from a single physician, filling 3% of the total morphine milligram equivalents (MME) dispensed. In contrast, 11% received opioid prescriptions from more than five physicians, filling 85% of all MME dispensed. There were 9008 physicians who prescribed opioids in 2010, and 15,486 in 2020. The percentage of physicians prescribing opioids to more than 50 patients/year increased from 7% in 2010 to 12% in 2020. The proportion of MMEs prescribed by those physicians increased from 36% in 2010 to 81% in 2020.

**Conclusion:**

We found that a small number of patients received the great majority of opioids, and a small number of physicians prescribed the great majority of opioids. Based on our results, we suggest policy options that would have minimal impact on most patients and most prescribers but would make a meaningful contribution to limiting opioid overprescription.

## Introduction

Opioid use has risen in Israel over the last decades. Attention to this issue increased when a report was published, suggesting levels of opioid usage comparable to those in the United States [1]. The rise in opioid prescription rates was confirmed in several follow-up publications [2–6]. The Israeli Ministry of Health convened committees to address this topic and issued requirements for healthcare providers to take steps to promote the monitoring and regulation of opioid prescriptions. Specific strategies are not established at the national level; rather, health maintenance organizations and hospitals are required to devise their own internal action plans [7].

We have previously shown that much of the opioids prescribed to non-oncological patients are received by a small number of patients, who usually receive high doses [5]. We sought to further examine this phenomenon in terms of which physicians are involved in the prescribing process. This includes whether patients received their prescription from one, several, or many physicians, and the extent to which physicians prescribed opioids to few or many patients within Clalit Health Services (CHS), Israel’s largest health maintenance organization. We examined increased opioid use through several different approaches: per patient—counts of physicians who prescribed opioids, and whether the prescribing physicians were their primary-care providers; per physicians—counts of physicians who prescribed opioids to large numbers of patients. Through these complementary analyses, we sought to understand the increased use of prescription opioids in the patient population managed by CHS.

## Methods

We conducted a historical cohort analysis using a data repository that included all adult patients in CHS who were prescribed opioids between 2010 and 2020. We excluded patients with oncologic and hemato-oncological diagnoses. The study was authorized by the CHS ethics institutional review board (COM1-159-19).

The data presented in this report were derived from CHS using the Clalit Research Data Sharing Platform powered by MDClone (https://wwwmdclone.com). CHS is the largest healthcare provider in Israel, serving approximately 51% of the population. All CHS members aged 18+, who had at least one opioid prescription filled between 2010 and 2020 and who were alive after December 31st, 2010, were included in the analysis. Opioids were classified according to ATC level opioid ingredients (buprenorphine, fentanyl, morphine, oxycodone, and tramadol). Codeine was excluded, as its use was both common and stable with no effect on larger trends, along with propoxyphene and pethidine, which were prescribed infrequently. Medication-assisted treatment with methadone and buprenorphine (oral and sublingual) was not provided in the community-based healthcare system in CHS during the study period, but rather only in designated rehabilitation clinics (either government-run or privately licensed), and not included in our analysis. Morphine milligram equivalents (MME) for each filled prescription were calculated according to accepted methods [8].

All patients within CHS are registered to a single primary-care physician at all times as his/her designated primary physician. These are either family physicians (board-certified) or general practitioners. In either case, these physicians are routinely informed of their assigned patients' consultations, laboratory and test results, and hospitalizations. Throughout this paper, we refer to physicians who have patients registered with them as “primary care physicians”, and differentiate between their prescribing to patients who are registered with them and to patients who are not. In contrast, there are consulting physicians (orthopedic specialists, surgeons, endocrinologists, etc.), as well as other general practitioners who work in clinics and to whom patients are not registered. Hospital-based physicians do not have patients registered with them.

We had de-identified unique serial codes for all physicians to whom patients were registered. The same coding system was used to provide unique identification of the physicians who prescribed the filled prescriptions, some of whom were primary-care physicians and others who were not. Thereby, we could identify whether the prescribing physicians for each prescription were the primary care physicians with whom the patient was registered at the time of prescription, other primary-care physicians (physicians with whom other patients were registered), or other non-primary-care physicians.

We examined the number of physicians from whom each patient received opioids and the number of physicians to whom each patient was registered during the study period, since some patients changed registration one or more times to different physician(s). We also assessed the difference in opioid prescription based on whether the patient was registered with the prescribing physician or with another physician. Additionally, we present data showing the number of patients physicians prescribed to and the amount of filled MMEs prescribed by these physicians.

The analysis of patients and the number of opioid prescribing physicians was repeated and limited to include only patients who received more than 1350 MMEs in total. This is the equivalent of three months of oxycodone 10 mg per day (15 MME daily). This is an arbitrary cut-point chosen to represent a patient who had at least received a non-trivial dose of opioids over a meaningful period of time. Still, admittedly, it may omit patients who received higher doses over shorter periods.

In this report, we present descriptive results, either crude numbers and percentages or rates.

## Results

During the study period, 825,584 adult patients filled 5,501,714 opioid prescriptions (Table 1). Half of the patients received opioid prescriptions from a single physician, and 5% from six or more physicians.

**Table 1:** Number of patients, prescriptions, and filled MMEs per number of prescribing physicians and patient-registered physicians

Number of prescribing physicians	Number of patients	Prescriptions (in thousands)				MME (in millions)			
		Total	Fent	Oxy	Tram	Total	Fent	Oxy	Tram
1	466,225 (56%)	785.7 (14%)	12.6 (4%)	119.9 (8%)	631.3 (19%)	173.7 (5%)	33 (3%)	44.1 (4%)	83.9 (13%)
2	176,055 (21%)	774.2 (14%)	20.1 (7%)	152.4 (10%)	566.6 (17%)	232 (7%)	58 (5%)	67 (6%)	93.7 (14%)
3	79,749 (10%)	678.5 (12%)	24.9 (8%)	158.3 (10%)	455.6 (14%)	270.7 (9%)	83.1 (7%)	85 (7%)	85.3 (13%)
4	40,714 (5%)	576.5 (11%)	26.6 (9%)	149.7 (9%)	361.5 (11%)	280 (9%)	93.9 (8%)	94.9 (8%)	74.5 (11%)
5	22,848 (3%)	492.8 (9%)	27.7 (9%)	139.9 (9%)	288.8 (9%)	289 (9%)	105.6 (9%)	100.6 (9%)	65.2 (10%)
6 or more*	39,957 (5%)	2,194 (40%)	186.6 (63%)	865.8 (55%)	986.9 (30%)	1900.8 (60%)	755.9 (67%)	770.6 (66%)	268.4 (40%)
*Number of primary-care physicians*									
1	680,419 (82%)	2,924.6 (53%)	122 (41%)	733.1 (46%)	1918.7 (58%)	1318 (42%)	433.6 (38%)	456.6 (39%)	354.8 (53%)
2	111,409 (13%)	1,375.2 (25%)	75 (25%)	403.6 (25%)	807.9 (25%)	791.2 (25%)	274.5 (24%)	296.2 (25%)	173.6 (26%)
3	23,870 (3%)	640.6 (12%)	46.3 (16%)	219.2 (14%)	329.1 (10%)	475.4 (15%)	179.9 (16%)	183.5 (16%)	81.6 (12%)
4	6542 (1%)	297.1 (5%)	27.3 (9%)	113 (7%)	134.7 (4%)	268.9 (8%)	115.2 (10%)	103 (9%)	36.6 (5%)
5	2039 (0.2%)	129.5 (2%)	12.1 (4%)	51.2 (3%)	56.5 (2%)	129.6 (4%)	49.2 (4%)	57.4 (5%)	16.1 (2%)
6 or more**	1269 (0.2%)	134.6 (2%)	15.9 (5%)	65.8 (4%)	44.6 (1%)	163.1 (5%)	77 (7%)	65.5 (6%)	13.6 (2%)

^*^There were 33,505 (4.1%) patients who received opioid prescriptions from 6 to 10 physicians, 6399 (0.8%) who received prescriptions from 11 to 30 different physicians, 49 (0.01%) who received prescriptions from 31 to 60 physicians, and 4 (0.0004%) who received prescriptions from 61 to 94 physicians

^**^There were 743 (0.09%) patients who were registered to a total of six physicians during the study period. An even smaller number (296, or 0.04%) were registered to seven, 129 (0.02%) to eight, 62 (0.007%) to nine, 13 (0.001%) to ten, 10 (0.001%) to eleven, three (0.0003%) to twelve, nine (0.001%) to thirteen, and one patient each (0.0001%) was registered to 14, 16, 19, and 31 physicians, respectively

Patients who received opioid prescriptions from six or more physicians (5% of the total cohort) were prescribed a greater proportion of prescriptions (40%) and the majority of MME (60%). For the most potent opioid, fentanyl, only 4.2% of prescriptions and 2.9% of MME were filled by patients who received their prescriptions only from a single physician. The respective estimates for oxycodone were 7.6% and 3.8%. In contrast, 63% of fentanyl prescriptions and 67% of fentanyl MME went to patients who received prescriptions from six or more physicians. With oxycodone, these numbers were 55% and 66% (Table 1).

To assess the results presented in Table 1 and exclude patients who received short courses, we repeated the analysis, including 130,685 patients (16% of the original study cohort) who received more than 1350 MMEs in total. Among these patients, 10% received their opioids from only one physician, 17% from two, 18% from three, 15% from four, 12% from five, and 28% from six or more. In total, they were 16% of all patients. Again, the most notable findings are that only 4% fentanyl was prescribed to patients by a single physician, and that 28% of patients who received fentanyl were prescribed 54% of prescriptions and 65% of total MME (Table 2).

**Table 2:** Number of patients, prescriptions, and filled MMEs per number of prescribing physicians and patient-registered physicians among patients who filled more than 1350 MMEs between 2010 and 2020

Number of prescribing physicians	Number of patients	Prescriptions (in thousands)				MME (in millions)			
		Total	Fent	Oxy	Tram	Total	Fent	Oxy	Tram
1	13,137 (10.1%)	156.3 (4%)	10.6 (4%)	41.7 (3%)	93 (5%)	87.5 (3%)	32.1 (3%)	26.7 (2%)	23.7 (5%)
2	21,663 (16.6%)	320.2 (8%)	18.8 (6%)	88.9 (6%)	188.1 (9%)	168.6 (6%)	57.4 (5%)	53.1 (5%)	46.9 (9%)
3	23,332 (17.9%)	427.6 (11%)	24.4 (8%)	122.7 (9%)	247.5 (12%)	236.4 (8%)	82.9 (7%)	77.6 (7%)	60 (12%)
4	20,203 (15.5%)	456.1 (11%)	26.4 (9%)	133.8 (10%)	260.9 (13%)	264.2 (9%)	93.8 (8%)	91.7 (8%)	62.7 (12%)
5	15,751 (12.1%)	442 (11%)	27.6 (9%)	133.8 (10%)	245.6 (12%)	282.7 (10%)	105.6 (9%)	99.4 (9%)	60.3 (12%)
6 or more	36,599 (28%)	2164.5 (54%)	186.6 (63%)	862.7 (62%)	961.2 (48%)	1897.4 (65%)	755.9 (67%)	770.1 (69%)	265.6 (51%)

There were 5599 primary-care physicians in CHS whose patients filled opioid prescriptions. Among primary-care physicians, 1382 (25%) never prescribed any opioids themselves. However, the majority of filled opioid prescriptions and filled MMEs were prescribed by primary-care physicians, although not necessarily to their own registered patients (Table 3).

**Table 3:** Opioid prescriptions written by physicians to whom the patients were and were not registered to

Written by primary-care physicians	Prescriptions filled (in 100,000s)				MMEs (in Millions)			
	Total	Fentanyl	Oxycodone	Tramdol	Total	Fentanyl	Oxycodone	Tramadol
Yes	35.8 (64%)	2.1 (72%)	11.3 (70%)	20.1 (60%)	2.3 (72%)	0.85 (74.2%)	0.88 (74%)	0.45 (65%)
No	20.1 (36%)	0.86 (28%)	0.5 (30%)	13.4 (40%)	0.88 (28%)	0.29 (25.8%)	0.3 (26%)	0.24 (35%)

*MMEs* Morphine milligram equivalents

Primary-care physicians who prescribed opioids had, on average, more registered patients who filled opioid prescriptions than primary-care physicians who did not prescribe any opioids. They even had more registered patients who received opioids from physicians who were not their primary-care physicians (Table 4). Relatedly, patients registered with primary-care physicians who prescribed opioids received, on average, more MMEs from their primary-care physicians than from other physicians.

**Table 4:** Comparison of opioid prescriptions between patients registered to opioid-prescribing primary care physicians and those registered to non-prescribing physicians

	Patients registered with non-prescribing primary care physicians		Patients registered with opioid prescribing primary care physicians	
	Received from their own physician	Received from other physicians	Received from their own physician	Received from other physicians
*Average number of patients registered per physician*				
Any opioid	0	60	295	147
Fentanyl	0	2	9	4
Oxycodone	0	14	75	32
Tramadol	0	52	250	126
*MME per patient*				
Any opioid	0	2,039	1,835	917
Fentanyl	0	17,927	19,612	11,075
Oxycodone	0	2,966	2665	1,408
Tramadol	0	670	484	289

**Fig. 1:**
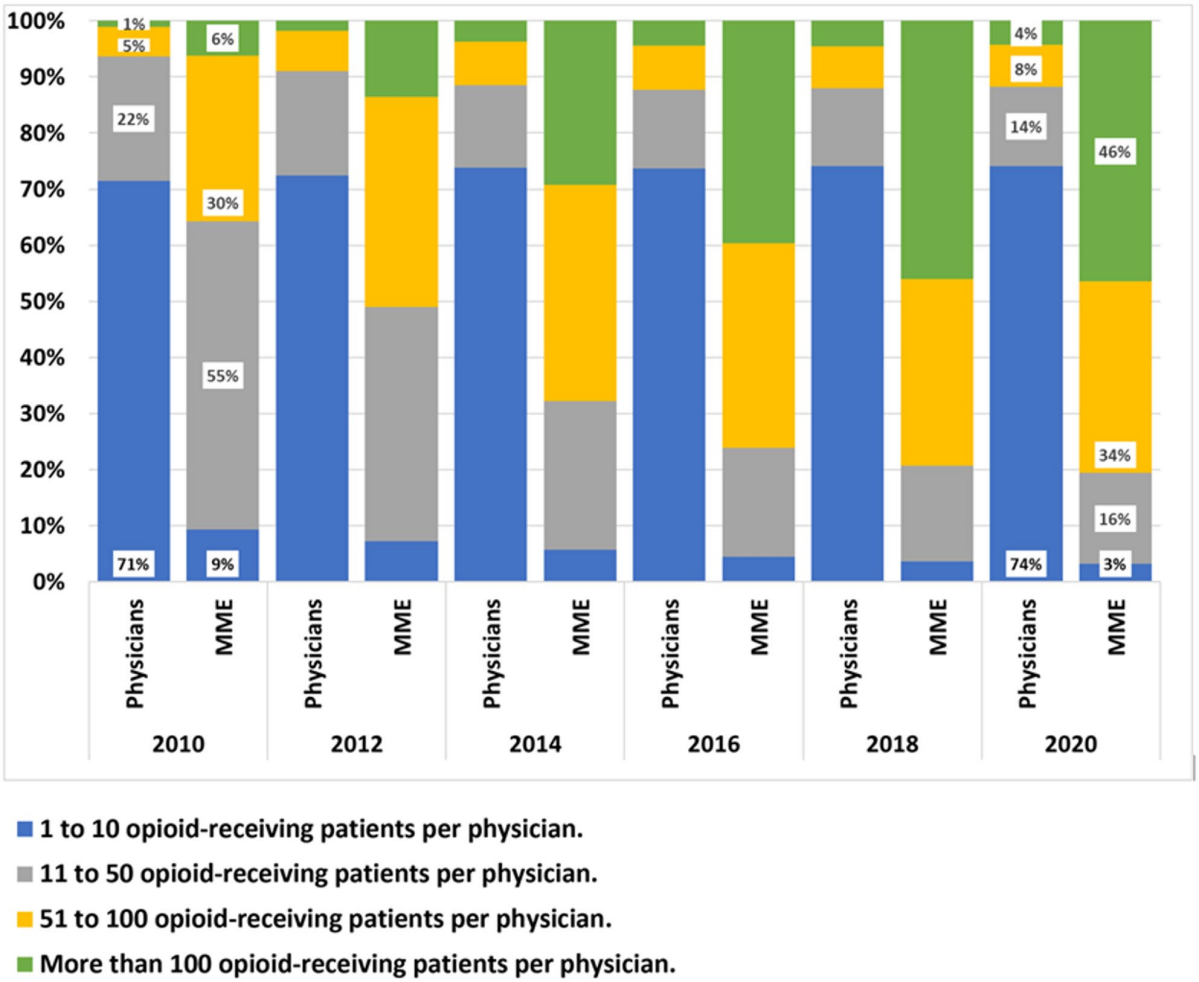
Proportion of physicians who prescribed opioids to few or many patients and proportion of the total morphine-milligram equivalents prescribed per group. The figure illustrates the proportions of physicians in the years 2010, 2012, 2014, 2016, 2018, and 2020, from left to right. In each year, there are two columns: the left column presents the proportion of physicians who prescribed opioids to 1 to 10 patients in that year (blue), 11 to 50 patients in that year (grey), 51 to 100 patients in that year (yellow) or more than 100 patients (green); The right column for each year presents the respective proportion of filled opioid MMEs for each physician group. For example, in 2010, 71% of physicians who prescribed opioids did so to 1 to 10 patients. These filled prescriptions accounted for 9% of total MMEs in that year. In 2020, the proportion of physicians who prescribed to 1 to 10 patients was 74% (similar to 2010), and the filled prescriptions accounted for 3% of total MMEs. In contrast, in 2010, 1% of physicians who prescribed opioids did so to more than 100 patients during that year; these prescriptions accounted for 6% of total filled MMEs. In 2020, 4% of opioid prescribing physicians prescribed to more than 100 patients, and these prescriptions accounted for 46% of total filled MMEs

The total number of physicians (primary care and other) who prescribed opioids in 2010 was 9008, of whom 4138 (46%) prescribed opioids to a single patient, 2,301 (26%) prescribed to two to ten patients, and 1994 (22%) prescribed to 11 to 50 patients. Through 2020, the absolute number of physicians who prescribed any opioids increased to 15,486, though the relative proportions were similar: 7352 (47%) prescribed opioids to a single patient, 4126 (27%) to 2–10 patients, and 2184 (14%) to 11–50 patients. The percentage of physicians prescribing opioids to up to 50 patients in a year remained relatively stable, 93% in 2010 and 88% in 2020. However, the proportion of MMEs prescribed by those physicians decreased—in 2010, it was 64% of total prescribed MMEs, and by 2020, it was only 19% of total MMEs prescribed (Fig. 1).

Examining patients who had changed registered physicians the most, 101 patients had at least 9 registered physicians. This group of 101 patients each received opioid prescriptions from between 10 and 94 different physicians. A total of 1453 physicians prescribed opioids to these 101 patients: 1022 physicians prescribed to one of these patients, 262 physicians prescribed to two of these patients, 91 physicians prescribed to three of these patients, 46 to four, and 19 to five patients. A single physician prescribed for 51 of these patients. Four primary-care physicians were each appointed to seven of these 101 patients, two physicians were each appointed to eight of them, and one physician was appointed to 20 patients. In total, 730 physicians were the primary-care physicians of these patients at some point, and 566 had a single registered patient from this group. This suggests both that these patients changed their registered physicians frequently and that a relatively small group of physicians was far more likely to be registered to them than would be expected based on chance alone.

## Discussion

We undertook this analysis of data from CHS to better understand opioid prescription patterns in Israel and to propose policy amendments directed at better opioid prescription practices. Our findings suggest that relatively few patients and relatively few doctors account for the great majority of opioids, both in terms of the number of prescriptions and MME. In addition, we noted that patients who change their primary-care physician frequently account for a greatly disproportionate share of the opioids prescribed. Though the percentage of physicians who prescribed opioids to multiple patients was low, they accounted for an increasing proportion of dispensed MME in the later years of the study.

Until 2020, fentanyl, an opioid that probably requires the closest supervision, may have been the most loosely supervised [9]. Most patients who received fentanyl received it from multiple physicians. Less than 2% of fentanyl prescriptions, and less than 1% of fentanyl MME, were prescribed by a single physician.

In a previous study, we demonstrated that the great majority of patients who received any opioid received them in modest amounts [5]. Examining our current findings, we see that only a few patients and a few physicians accounted for the great majority of what was dispensed. This was related to the number of physicians who prescribed for each patient. Of our study patients, 88% received opioids from up to 5 physicians, and about 0.2% received prescriptions from more than 30 physicians. Most patients did not have more than a single registered physician during the study period.

Our results point to a great need for policy initiatives to improve opioid prescribing in Israel, and also offer tantalizing hints as to what levers could be used to effect meaningful change. Historically, in Israel, regulation of opioid prescribing has been minimal, and it was not regarded as a major problem. Until 2020, there were no specific training or qualification requirements for opioid prescriptions, and any licensed physician could (and still can) prescribe any opioid at any dosage.

The four health funds (“kupot cholim”) have the most direct control over care provision. The health funds already have programs in place to monitor and limit opioid prescribing, but our report suggests that they should devote additional resources to such monitoring and also suggests some of the parameters they may wish to monitor. In particular, focusing on the disproportionate opioid prescribing of a few physicians and opioid receipt of a few patients may be more important than hiding these numbers in a larger average. Our report also suggests the possibility of further actions that would be possible concerning outlier prescribers or recipients of opioids. Limitations on opioid prescribing can include limiting prescriptions to designated physicians, directed interventions to improve prescribing among outlier physicians, and limitations on potent opioids such as fentanyl. There could also be softer measures such as academic detailing, which in turn requires excellent data and monitoring [10].

There are other bodies that have an important role to play here. Our report, which represents a cooperative effort between Israel’s universities and the health funds, is typical of the role often played by universities. Universities have little direct responsibility for care delivery or setting policy, but university-driven research can point the way toward monitoring and policy changes that may have been overlooked until now. The authorship group of this paper have connections at universities and at health funds, meaning that they have access both to the new ideas provided by university settings and to the more direct responsibility for setting policy. Universities will need to continue in this role of shedding light on problems not heretofore recognized.

The Israel Medical Association (IMA) and its specialty societies have an important role to play here. These organizations do not set policy; however, they publish professional guidelines and ethical standards, and they are politically powerful and can certainly stand in the way or help propagate policy changes if they wish to do so. Our report implies that the autonomy of the great majority of physicians could be relatively unaffected by changes that could nevertheless have a profound impact on opioid prescribing. It is our hope that the IMA will work together with others to promote the necessary changes. Indeed, professional societies can have a very positive effect as well, insofar as they can provide powerful statements of shared norms and responsibilities for physicians, and we hope that the IMA will take such a leadership role.

Finally, the Ministry of Health may be involved in creating new regulations for opioid prescribing, perhaps create and require opioid prescription licenses. We propose that such actions be considered in consultation with the health funds, and not over their objections. At its best, the Ministry of Health may be involved in giving the health funds the necessary authority to do what is necessary if they identify an aspect of opioid prescribing where they would need additional tools to improve prescribing.

While our results have great importance for improving health services delivery in Israel, our results also have great relevance outside Israel. This particular way of looking at opioid prescribing patterns—looking at which doctors are registered to which patients, and who is prescribing to whom—is relatively unique in the literature, and was extremely revealing both regarding what is going on in our system and how it might be addressed through policy changes. We would recommend this sort of analysis as very much worth performing to better understand opioid prescribing patterns in any system of care, particularly one where patients are formally assigned to a primary care doctor, which is true in most systems.

One way to frame our results, at least in part, is through the lens of doctor shopping. Doctor shopping is usually based on measurable behavior rather than mental process, most often using combinations of numbers of prescribers and dispensing pharmacies over periods ranging from 90 days to 2 years [11]. Over time, the prevalence and rates of doctor shopping have been noted to change, sometimes declining [12, 13] or rising [14, 15], depending on the definitions used and the period under study. Rates have been reported per population size [12], or as a proportion of opioid prescribed patients [15–17], or of all prescriptions [13]. The percentage of doctor shoppers out of opioid prescribers has been reported to be between 1% and 7.5% in various studies [15–17].

Our study suggests that some of our patients engaged in doctor shopping, and that those patients were receiving a massively disproportionate share of the opioid prescriptions in our dataset. Our results echo a previous report, which found that patients defined as doctor-shoppers receive a large proportion of the opioids prescribed by their primary doctor. A study of California’s de-identified Controlled Substance Utilization Review and Evaluation System (CURES) database from the years 2008–2015 found that doctor shoppers received 23% of prescriptions and 9% of their total MME from one-time prescribers, compared with 28% of their prescriptions and 40% of their total MME from their primary provider [16]. We did not find studies that examined, as we did, the relative proportion of opioid consumption associated with doctor shopping and multiple prescribers. In this respect, our findings are novel, though we believe they are not unique, and likely reproducible in other healthcare systems.

Some have suggested that patients who have multiple prescribers might be seeking genuine care, and may be consulting multiple doctors to pursue a workup or a diagnosis [18]. However, whether their care-seeking is genuine or deceitful, any patient who is prescribed opioids over a long period can develop opioid use disorder, which can progress to full addiction, increased risk of overdose, and early mortality.

. Our report highlights the importance of (1) enhanced capacity for real-time monitoring, with a focus on disproportionate prescribers and disproportionate recipients of opioids; (2) enhanced capacity for academic detailing based on such monitoring, with an emphasis on rapid responses; (3) stricter limits on who can prescribe fentanyl and how; (4) a need for periodic review and approval for opioids that are prescribed beyond a certain threshold of dose or duration; and (5) potential revocation of opioid prescribing for physicians who continue to engage in problematic prescribing despite these efforts. Some other options that may be considered include (1) flagging prescriptions to a single patient by multiple physicians; (2) requiring all opioids to be prescribed to each patient only by a single physician; and (3) limiting the ability of patients receiving ongoing opioid prescriptions to change primary physicians, or requiring administrative review to approve such a change.

However, there could also be pitfalls or unintended consequences with such policy approaches. It will be especially important to have ongoing evaluation of the impact of such changes to ensure that they do not unduly burden prescribers or patients, or make it difficult to access needed health care. Furthermore, patients might choose to move between healthcare providers if they identify less stringent supervision of opioid dispensation—creating a marketing dilemma for the care providers. This is why we think consideration of oversight for switching primary doctors among patients receiving ongoing opioid therapy may be warranted. Finally, patient advocacy groups might perceive limitation of access to pain relief medication as a paternalistic strategy, rather than a benevolent one, incurring additional pressure on providers through the courts and media. This issue can be anticipated and plans should be made in advance to address it proactively with strong communication strategies. Finally, though we focus on primary-care physicians, their registered patients, and reduced mobility, we should also note that about a third of opioid prescriptions and MMEs were prescribed by community physicians who were not primary-care physicians. Carefully devised prescription limitations could be introduced, while remaining mindful that acute short-term care should not be impeded.

Our report has important limitations. First, our data did not include private prescriptions dispensed in a private setting. We assume that this is not widespread, since the structure of Israeli healthcare would discourage such patterns of care. Second, our data cannot differentiate between patients who consume opioids and those who divert them. While the two would look the same in our data, from a policy point of view, patients who are consuming opioids and patients who are diverting them may require a different approach. Finally, our data lack certain details that might have given us insight into what disorders are being treated, and the stated clinical reasoning behind each prescription. While future studies should delve into such details, our study does provide a valuable view of the broad picture of what is going on in CHS, and by extension, the Israeli population, and as such, offers important insights that would be complemented by a more micro view in additional studies. Our rather novel approach to understanding who is prescribing to whom is also one that could be broadly applied in most systems.

## Conclusions

We analyzed data from Clalit Health Services to better understand the precipitous increase in opioid prescriptions between 2010–2020. We found that a small number of patients are receiving the great majority of opioids, and a small number of physicians are prescribing the great majority. Our results imply that policies could be designed that would have a minimal impact on most patients and most prescribers but would make a meaningful contribution to bringing opioid prescriptions under better control. We detail our recommendations for how Israel’s health funds could change their policies to improve opioid prescribing in light of our findings here, and how other stakeholders can help with this effort. Our results also suggest an approach that could be helpful outside Israel, in terms of focusing additional attention on relatively few prescribers and patients who account for the great majority of opioid prescribing.

## Data Availability

No datasets were generated or analysed during the current study.
